# Noncytotoxic and Antitumour-Promoting Activities of Garcinia Acid Esters from *Garcinia atroviridis* Griff. ex T. Anders (Guttiferae)

**DOI:** 10.1155/2012/829814

**Published:** 2012-05-28

**Authors:** Mukram M. Mackeen, Lim Y. Mooi, Mohidin Amran, Nashriyah Mat, Nordin H. Lajis, Abdul M. Ali

**Affiliations:** ^1^Department of Cell and Molecular Biology, Faculty of Biotechnology and Biomolecular Sciences, Universiti Putra Malaysia, Selangor, 43400 Serdang, Malaysia; ^2^Chemistry Research Laboratory, Department of Chemistry, University of Oxford, Mansfield Road, Oxford OX1 3TA, UK; ^3^Department of Preclinical Sciences, Universiti Tunku Abdul Rahman, Lot PT21144, Jalan Sungai Long, Selangor, 43000 Kajang, Malaysia; ^4^Institute of Bioscience, Universiti Putra Malaysia, Selangor, 43400 Serdang, Malaysia; ^5^Sime Darby R&D Centre, Carey Island, Lot 2664, Jalan Pulau Carey, Selangor, 42960 Carey Island, Malaysia; ^6^Faculty of Agriculture and Biotechnology, Universiti Sultan Zainal Abidin, Gong Badak Campus, Terengganu, 21300 Kuala Terengganu, Malaysia

## Abstract

The *in vitro* antitumour-promoting, cytotoxic, and antioxidant activities of two ester derivatives of garcinia acid, that is, 2-(butoxycarbonylmethyl)-3-butoxycarbonyl-2-hydroxy-3-propanolide (**1**) and 1′,1′′-dibutyl methyl hydroxycitrate (**2**), that had been previously isolated from the fruits of *Garcinia atroviridis* Griff. ex T. Anders (Guttiferae), were examined. Based on the inhibition of Epstein-Barr virus early antigen (EBV-EA) activation, compound **1** (IC_50_: 70 **μ**M) showed much higher (8-fold) antitumour-promoting activity than compound **2** (IC_50_: 560 **μ**M). In addition, both compounds were nontoxic towards CEM-SS (human T-lymphoblastic leukemia) cells (CD_50_: >100 **μ**M), Raji (human B-lymphoblastoid) cells (CD_50_: >600 **μ**M), and brine shrimp (LD_50_: >300 **μ**M). Although the antitumour-promoting activity of compound **1** is moderate compared with the known antitumour promoter genistein, its non-toxicity suggests the potential of compound **1** and related structures as chemopreventive agents. The weak antioxidant activity displayed by both compounds also suggested that the primary antitumour-promoting mechanism of compound **1** did not involve oxidative-stress quenching.

## 1. Introduction

*Garcinia atroviridis* Griff. ex T. Anders (Guttiferae) is a medium-sized fruit tree endemic to Peninsular Malaysia. This species grows wild throughout Peninsular Malaysia but is also widely cultivated especially in the northern states owing to its economic and medicinal values. In folkloric medicine, *G. atroviridis* is used as a post-partum medication and to treat earache, throat irritation, cough, dandruff, and stomach pains associated with pregnancy. Sun-dried slices of the fruits are commercially available and are popularly used as a seasoning in curries, sour relish and also for dressing fish. The young leaves are also used for culinary purposes and as a traditional vegetable [1].

**Figure 1:**
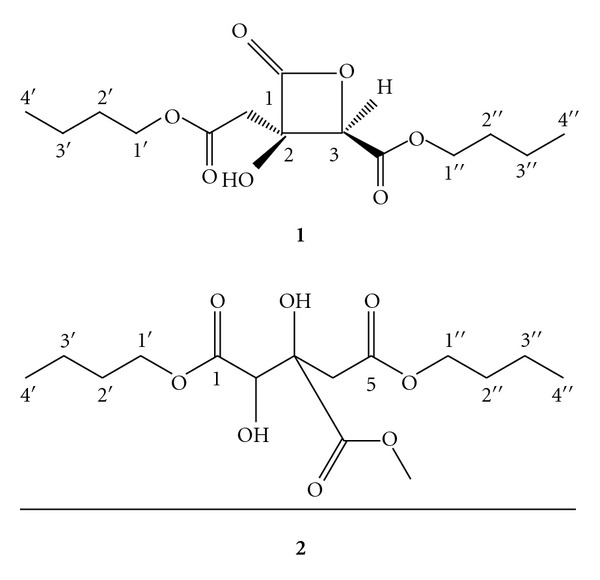
The structures of the compound **1** (2-(butoxycarbonylmethyl)-3-butoxycarbonyl-2-hydroxy-3-propanolide) and compound **2 (**1′,1′′-dibutyl methyl hydroxycitrate).

The occurrence of xanthones, benzophenones, and biflavonoids is common in the *Garcinia *genus [2]. To date, the metabolites that have been isolated from *G. atroviridis* are atroviridin (xanthone); atrovirinone and 4-methylhydroatrovirinone (prenylated quinones); atrovirisidone and atrovirisidone B (prenylated depsidones); along with the known metabolites morelloflavone, fukugiside, naringenin, 3,8′′-binaringenin, 14-*cis*-docosenoic acid, garcinia acid (same as (−)-hydroxycitric acid) and its *γ*-lactone [3–7]. Garcinia acid, which has been primarily obtained from the *Garcinia *genus, is an effective inhibitor of lipogenesis with commercial and clinical applications [8]. Previously, two garcinia acid esters, namely, 2-(butoxycarbonylmethyl)-3-butoxycarbonyl-2-hydroxy-3-propanolide and 1′,1′′-dibutyl methyl hydroxycitrate (Figure 1) had been isolated from the fruits of *G. atroviridis,* guided by antifungal activity against *Cladosporium herbarum* [1, 9]. In this paper, we reported the antitumour-promoting, antioxidant and cytotoxic activities of both these compounds.

## 2. Materials and Methods

### 2.1. Source of Garcinia Acid Esters

The two esters derivatives of garcinia acid, 2-(butoxycarbonylmethyl)-3-butoxycarbonyl-2-hydroxy-3-propanolide **(1)** and 1′,1′′-dibutyl methyl hydroxycitrate **(2)**, were available from our previous isolation of these compounds from *Garcinia atroviridis* Griff. ex T. Anders (Guttiferae). Details of the isolation and identification were described in [1].

### 2.2. Culture of Cells

The CEM-SS (human T-lymphoblastic leukemia) cell line was obtained from the National Cancer Institute, USA, and the Raji (human B-lymphoblastoid) cell line was provided by Prof. K. Koshimizu, Kinki University, Japan. Cells were cultured in RPMI-1640 (Sigma, USA) medium with 10% v/v foetal calf serum (Sera Lab, UK), 100 IU/mL penicillin (Sigma, USA) and 100 *μ*g/mL streptomycin (Sigma, USA) as a complete growth medium (CGM). Cells were maintained in 25 cm^2^ flask with 10 mL of CGM at 37°C with 5% CO_2_. Every three days the cells were subcultured by splitting the culture with fresh CGM at a ratio of 1 : 4 [10].

### 2.3. MTT Cytotoxicity Assay

 Cytotoxicity was determined using the MTT assay as reported by Ali et al. [10]. Varying concentrations of the test compounds were prepared from the stock solutions by serial dilution in RPMI-1640 to give a volume of 100 *μ*L in each well of a microtiter plate (96-well) as described before. Each well was filled with 100 *μ*l of CEM-SS cell suspension in CGM at 1-2 × 10^5^ cells/mL. Controls that contained only CEM-SS cells were included for each sample. The assay for each concentration of extract was performed in triplicate and the culture plates were kept at 37°C with 5% (v/v) CO_2_ for three days. After 72 h of incubation, 100 *μ*L of medium was removed from each well. Subsequently, 20 *μ*L of 0.5% w/v MTT (Sigma, USA) dissolved in phosphate buffer saline was added to each well and allowed to incubate for further 4 h. After 4 h of incubation, 100 *μ*L of 1 N hydrochloric acid : isopropanol (1 : 24) was added to each well and vigorously mixed to dissolve the formazan crystals. Absorbance values at 550 nm were measured with a microplate reader (Bio Tek EL 340, USA) after background subtraction at 630 nm. Cytotoxicity was expressed as CD_50_, that is, the concentration to reduce the absorbance of treated cells by 50% with reference to the control (untreated cells).

### 2.4. Brine Shrimp Lethality/Toxicity Assay

Brine shrimp (*Artemia saline* Leach) eggs (Gold Eagle, USA) were placed in a hatching tank containing sea water for 48 h [11]. Each compound (6 *μ*mol) was dissolved in 0.2 mL methylene chloride:methanol (1 : 1) to prepare a stock solution of 30 mM. From the stock solutions, 50 *μ*L was transferred to different vials and allowed to evaporate. After evaporation, 5 mL of brine was added to each vial in triplicate to prepare a preliminary test concentration corresponding to 300 *μ*M. Ten shrimp nauplii were added to each vial (30 shrimps per concentration). The number of survivors out of 30 shrimps per concentration was recorded.

### 2.5. *In Vitro* Antitumour-Promoting Assay

The inhibition of Epstein-Barr virus (EBV) activation was used to evaluate *in vitro* antitumour-promoting activity [12]. Raji cells were incubated for 48 h at 37°C under 5% CO_2_ in 1 mL (~5 × 10^5^ cells) of RPMI-1640 medium containing 3 mM sodium *n*-butyrate (Nacarai Tesque, Japan), 50 nM phorbol 12-myristate 13-acetate (PMA) (Sigma, USA) and the test compounds (600 *μ*M) per well of a 24-well plate. Conventional indirect immunofluorescence staining of early antigen (EA) with high-titre EA-positive serum (EA titre 1 : 1,280) obtained from nasopharyngeal carcinoma (NPC) patients (1 : 20 serum dilution) followed by a 1 : 30 dilution of fluorescein-isothiocyanate-(FITC-)labeled IgG (Sigma, USA) was used to measure EBV activation. The ratio of EA-induced cells to the control experiments only with sodium *n*-butyrate and PMA was around 50%. The test compounds showing antitumour-promoting activity were serially diluted (2-fold) to determine the IC_50_ values.

### 2.6. Antioxidant Assays

#### 2.6.1. Ferric Thiocyanate (FTC) Method

A screw-cap vial (*∅* 38 × 75 mm) containing a mixture of 4 mg (4 mL) of a sample (final concentration, 0.02%) in 99.5% ethanol, 4.1 mL of 2.5% linoleic acid (TCL, Japan) in 99.5% ethanol, 8.0 mL of 0.05 M phosphate buffer (pH 7.0), and 3.9 mL of water was placed in an oven at 40°C in the dark [13]. To 0.1 mL of this mixture, 9.7 mL of 75% (v/v) ethanol and 0.1 mL of 30% ammonium thiocyanate (Wako, Japan) were added. Three minutes after the addition of 0.1 mL of 2 × 10^−2 ^M ferrous chloride (Merck, Germany) in 3.5% hydrochloric acid to the reaction mixture, the absorbance was measured at 500 nm (Spectronic 20D+, USA) each 24 h until one day after absorbance of the control reached its maximum value. Antioxidant activity was represented by the absorbance readings on the final day of the assay (7th day) (average of triplicates).

#### 2.6.2. Thiobarbituric Acid (TBA) Method

Two mL of 20% trichloroacetic acid (Fluka, Switzerland) and 2 mL of 0.67% TBA (Wako, Japan) solutions were added to 2.0 mL from the mixture (containing sample) prepared in the FTC method [13]. This mixture was kept in a water bath (100°C) for 10 min and, after cooling to room temperature, was centrifuged at 3000 rpm for 20 min. Antioxidant activity was based on the absorbance of the supernatant at 532 nm wavelength on the final day of the FTC assay (average of triplicates).

#### 2.6.3. Radical Scavenging Method

The methanol solution of 2,2-diphenyl-1-picrylhydrazyl hydrate (DPPH) (Aldrich Chem. Co., USA) radicals (1 mL, 0.3 mM) was added to each sample (1 mM, 0.5 mL) and was shaken vigorously [14]. The absorbance of each sample was measured at 517 nm after allowing it to stand for 30 min. Each sample (final concentration, 0.3 mM) was assayed in triplicate and averaged. Standard antioxidants, that is, butylated hydroxytoluene (BHT) and ascorbic acid, were used as positive controls.

## 3. Results and Discussion

In the antitumour-promoting assay, compound **1**, a *β*-lactone, strongly inhibited (IC_50_: 70 *μ*M) EBV activation compared with compound **2** (IC_50_: 560 *μ*M). The IC_50_ of **1** was almost five-fold higher than the value of the crude fruit extract (IC_50_: 97 *μ*g/mL). Compounds **1** and **2** were considered as nontoxic against CEM-SS and Raji cells (CD_50_: >100 *μ*M). Both compounds were also inactive (LC_50_: >300 *μ*M) in the brine shrimp lethality assay that is used as an *in vivo* surrogate model for cytotoxicity. The antitumour-promoting activity of **1** was noteworthy because of its noncytotoxicity although it did not inhibit EBV activation as strongly as the known antitumour-promoter, genistein (IC_50_: ~20 *μ*M), which is cytotoxic [15, 16]. Since inhibition towards EBV-EA activation has been demonstrated to closely parallel *in vivo* antitumour promotion [17], compound **1** has the potential as a chemopreventive agent.

**Figure 2:**
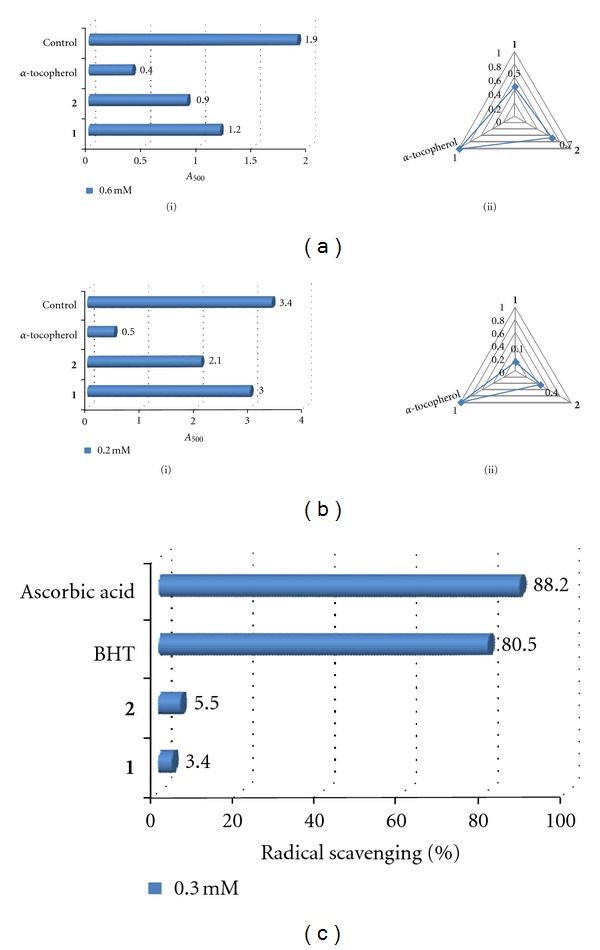
Antioxidant activities of compounds **1** and **2**. (a) FTC assay (i) absorbance at 500 nm, (ii) ratios of antioxidant values normalized to *α*-tocopherol. (b) TBA assay (i) absorbance at 532 nm, (ii) ratios of antioxidant values normalized to *α*-tocopherol. (c) DPPH assay. The sample test concentrations in (a) to (c) are 0.6 mM, 0.2 mM, and 0.3 mM, respectively. Assays were carried out in triplicate and averaged (±0.1 absorbance units).

The antioxidant assays performed on both compounds showed weak activity that was slightly higher than the control. Compounds **2** and **1**, respectively, reduced oxidant activity by 36 and 11% in the FTC assay (Figure 2(a)), 53 and 37% in the TBA assay (Figure 2(b)), and 5.5 and 3.4% in the DPPH assay (Figure 2(c)) but were weaker than the positive control(s) especially in the radical scavenging assay. It appeared that both these compounds were more effective inhibitors against late-stage oxidation as evidenced by the stronger antioxidant activity in the TBA assay than the FTC assay. The low antioxidant activity of both compounds ruled out the influence of oxidative-stress quenching as the dominant mechanism of action responsible for the antitumour-promoting activity of the *β*-lactone, although this mechanism is closely associated with antitumour-promoting activity [18]. This was further confirmed by the recurring trend of **2** showing higher activity than **1 **in all the antioxidant assays whereas stronger antitumour-promoting activity was displayed by **1** than **2**.

## 4. Conclusions

Of the two new ester derivatives of garcinia acid, that is, 2-(butoxycarbonylmethyl)-3-butoxycarbonyl-2-hydroxy-3-propanolide (**1**) and 1′,1′′-dibutyl methyl hydroxycitrate (**2**), previously isolated from the fruits of *Garcinia atroviridis*, only compound **1** showed antitumour-promoting activity although both compounds were neither toxic against the cells tested and brine shrimp nor were particularly antioxidant. This suggested the potential of compound **1** and related structures as noncytotoxic chemopreventive agents.
